# Children With Autism Produce a Unique Pattern of EEG Microstates During an Eyes Closed Resting-State Condition

**DOI:** 10.3389/fnhum.2020.00288

**Published:** 2020-10-08

**Authors:** Sahana Nagabhushan Kalburgi, Allison P. Whitten, Alexandra P. Key, James W. Bodfish

**Affiliations:** ^1^Vanderbilt Brain Institute, Vanderbilt University, Nashville, TN, United States; ^2^Vanderbilt University Medical Center, Nashville, TN, United States; ^3^Vanderbilt Kennedy Center, Nashville, TN, United States; ^4^Department of Hearing and Speech Sciences, Vanderbilt University Medical Center, Nashville, TN, United States

**Keywords:** EEG, microstates, resting-state, autism spectrum disorders, salience network

## Abstract

Although fMRI studies have produced considerable evidence for differences in the spatial connectivity of resting-state brain networks in persons with autism spectrum disorder (ASD) relative to typically developing (TD) peers, little is known about the temporal dynamics of these brain networks in ASD. The aim of this study was to examine the EEG microstate architecture in children with ASD as compared to TD at rest in two separate conditions – eyes-closed (EC) and eyes-open (EO). EEG microstate analysis was performed on resting-state data of 13 ASD and 13 TD children matched on age, gender, and IQ. We found that children with ASD and TD peers produced topographically similar canonical microstates at rest. Group differences in the duration and frequency of these microstates were found primarily in the EC resting-state condition. In line with previous fMRI findings that have reported differences in spatial connectivity within the salience network (previously correlated with the activity of microstate C) in ASD, we found that the duration of activation of microstate C was increased, and the frequency of microstate C was decreased in ASD as compared to TD in EC resting-state. Functionally, these results may be reflective of alterations in interoceptive processes in ASD. These results suggest a unique pattern of EEG microstate architecture in ASD relative to TD during resting-states and also that EEG microstate parameters in ASD are susceptible to differences in resting-state conditions.

## Introduction

Numerous studies of functional connectivity in ASD using fMRI have examined the network-level brain alterations in ASD at rest (“resting-state”) compared to typically developing (TD) individuals (Hull et al., 2017). While there is no signature pattern in the network alterations in ASD, studies provide evidence for both over- and under-connectivity (Cherkassky et al., 2006; Hughes, 2007; Assaf et al., 2010; Di Martino et al., 2011; Abrams et al., 2013; Supekar et al., 2013; Tyszka et al., 2014; Cerliani et al., 2015; Chen S. et al., 2017; Tomasi and Volkow, 2019). A majority of resting-state fMRI studies in persons with ASD report aberrant connectivity within the salience network (SN) (Supekar et al., 2013; Nomi and Uddin, 2015; Abbott et al., 2016; Green et al., 2016; Chen H. et al., 2017; Damiani et al., 2018). Indeed, the specificity of this effect was demonstrated in one study that showed that 78% of their sample of ASD participants could be classified accurately based on fMRI SN connectivity alone (Uddin et al., 2013). In addition, the same study found that the degree of SN activity strongly correlated with the severity of repetitive behavior phenotype in ASD, suggesting that SN activity may be important for understanding specific aspects of the ASD phenotype.

Although considerable evidence exists in ASD regarding the structural characteristics of brain networks in general (Hull et al., 2017) and the SN in particular (Uddin et al., 2013; Abbott et al., 2016; Chen H. et al., 2017), much less is known about the temporal dynamics of these networks. Reports of longer fMRI dwell times (Bressler and Seth, 2011; Barnett and Seth, 2014; Yuan et al., 2016), i.e., the duration of activation of brain networks, have been shown to be associated with the severity of ASD symptoms (Watanabe and Rees, 2017; Rashid et al., 2018). Comparable dynamics were observed by King et al., 2018, who found prolonged temporal synchrony of functional networks in ASD (King et al., 2018). One study found fewer rapid transitions between brain networks in ASD compared to TD (Watanabe and Rees, 2017). Studies by Bernas et al., 2018 and Damiani et al., 2018 have confirmed the abnormal temporal structure of the resting-state SN in ASD (Bernas et al., 2018; Damiani et al., 2018). Although these studies have shed light on the temporal dynamics of brain network activity in ASD, they have been limited by the time resolution of the BOLD fMRI signal.

To overcome these limitations in time resolution, recent studies have used EEG microstates as an approach to study the temporal dynamics of brain networks (Michel and Koenig, 2018). An advantage of considering EEG microstates as a proxy for network activation is that their timescale coincides with the sub-second range of synchronous firing of large neural networks. EEG microstates create a global representation of a functional state and are defined by the topography of electric potentials of a multichannel electrode array (Strik and Lehmann, 1993). These microstate topographies remain quasi-stable for 80–120 ms before switching to different microstate topographies (Wackermann et al., 1993; Khanna et al., 2015). The transitions between microstates can represent sequential activation of different neural networks, and in this way the dynamic nature of the neural basis of cognitive activity can be measured (Milz et al., 2016; Seitzman et al., 2017). EEG microstate analysis is rich with parameters of potential neurophysiological relevance. These parameters include: (a) The average duration of each microstate, which is the average length of time a given microstate remains stable. (b) The frequency of each microstate, which is the average number of times per second that the microstate becomes dominant during the recording period. (c) The coverage of a microstate, which is the fraction of total recording time that the microstate is dominant. (d) The transition probabilities of a microstate, which indicate switching from one class to any other in a non-random sequence (Khanna et al., 2015).

Across studies, evidence has converged for four archetypal microstates that explain most of the global topographic variance in resting-state (Dierks et al., 1997; Koenig et al., 2002; Strelets et al., 2003; Brodbeck et al., 2012; Nishida et al., 2013; Drissi et al., 2016; Atluri et al., 2017; Seitzman et al., 2017; Sverak et al., 2017; Zappasodi et al., 2017). These have been labeled as A, B, C, and D based on their topographical configuration. Simultaneous fMRI-EEG studies as well as EEG source imaging studies suggest that these microstate classes correspond to the activity of previously identified networks for phonological processing, the visual network, the saliency network, and attention network, respectively (Britz et al., 2010; Custo et al., 2017). Alterations in the expression of these microstates have been observed in a variety of neuropsychiatric conditions including depression (Atluri et al., 2017), Alzheimer’s disease (Dierks et al., 1997; Strik et al., 1997; Nishida et al., 2013), frontotemporal dementia (Strik et al., 1997; Nishida et al., 2013; Grieder et al., 2016), stroke (Zappasodi et al., 2017), and schizophrenia (Merrin et al., 1990; Stevens et al., 1997; Strelets et al., 2003; Lehmann et al., 2005; Kikuchi et al., 2007; Kindler et al., 2011; Nishida et al., 2013; Tomescu et al., 2014; Sverak et al., 2017). However, to date, only two such studies have been done in persons with ASD. Jia and Yu (2019) found that resting-state microstate parameters differed in adolescents with ASD as compared to age-matched typical controls in data containing mixed periods of eyes-open (EO) and eyes-closed (EC) conditions with increased frequency and coverage of microstate B, decreased duration of microstate A, and decreased duration and coverage of microstate C. Given that the two eye conditions result in functional differences in microstate readouts (Seitzman et al., 2017), it is necessary to investigate these two conditions separately. In the second study, D’Croz-Baron et al., 2019 also found that several microstate parameters differed in ASD adults as compared to the control group in EC resting-state (D’Croz-Baron et al., 2019). This study found an increase in frequency and coverage of microstate B, and a decrease in the duration and coverage of microstate C. However, this study used the updated meta-criterion (Custo et al., 2017; Michel and Koenig, 2018) to select more than four classes of microstates so the results of the other microstate classes from these two studies are not comparable. Neither study examined the correlation between the microstate parameters and behavioral measures in ASD. Given the importance of controlling for methodological differences in how “resting-state” is operationalized during data collection, the purpose of this study was to investigate the resting-state EEG microstate architecture in children with ASD compared to typical controls under both EC and EO resting-state conditions. We hypothesized that the temporal dynamics of microstate C would be altered in ASD reflecting atypical SN activity.

## Materials and Methods

### Participants

Children with a diagnosis of ASD and TD children between the ages of 8–14 years were recruited to participate in this study. All participants’ parents provided written informed consent and received monetary compensation. The study was approved by the Vanderbilt University Institutional Review Board (140436). Demographic and clinical data on both groups is provided in Table 1.

**TABLE 1:** Participant demographics information.

**Characteristic**	**ASD (*n* = 13)**	**TD (*n* = 13)**	***t* value (*p*-value)**
	**Mean (SD)**	**Mean (SD)**	
Age (years)	9.7 (1.5)	10.4 (1.4)	1.23 (0.2306)
Gender	11 M/2 F	11 M/2 F	–
ABIQ Standard Score	101.3 (19.8)	102.1 (9.9)	0.13 (0.8974)
**Social Communication Questionnaire**			
Total	17.7 (6.2)	3.5 (2.5)	7.66 (<0.001)
**Social Responsiveness Scale**			
T-Score	73.0 (10.0)	–	–
**Repetitive Behavior Scale—Revised**			
Stereotyped Behavior	5.4 (2.2)	0.9 (1.7)	5.84 (<0.001)
Self-Injurious Behavior	2.6 (3.7)	0.2 (0.4)	2.325 (0.029)
Compulsive Behavior	5.2 (4.3)	0.1 (0.3)	4.27 (<0.001)
Ritualistic Behavior	7.2 (3.9)	0.1 (0.3)	7.04 (<0.001)
Total	34.9 (21.1)	1.8 (2)	5.63 (<0.001)
**Autism Diagnostic Observation Schedule**			
Social Affect + Restricted Repetitive Behavior	13.9 (4.3)	–	–
Total Severity	7.8 (1.6)	–	–

*ASD, Autism Spectrum Disorder; TD, typically developing; M, male; F, female.*

#### Children With ASD

A total of 25 children with ASD were recruited for this study through a variety of resources (email distribution service, ResearchMatch.org database, local autism support groups, and flyers posted in autism clinics). Participants were required to have an existing clinical diagnosis of ASD from a licensed clinician. All diagnoses were also confirmed during the study visit by the administration of the Autism Diagnostic Observation Schedule-2 [ADOS-2 (Lord et al., 2012)] by trained research personnel. Exclusion criteria included a history of comorbid psychiatric disorders, epilepsy (one child), brain abnormalities (one child: cortical dysplasia), ADOS-2 score below 7 (one child: ADOS score 4), left-handedness, insufficient or noisy EEG data (six children), and no data for either of the two eye conditions (three children). This resulted in the final sample of 13 children in the ASD group (two females) with a mean age of 9.7 years (*SD* = 1.5) and an age range between 8 years 2 months and 12 years 9 months. Within the final sample, 6 participants took various ADHD medications. 2 of the 6 also took SSRIs and 1 of the 6 also took risperidone. All six participants on medication were on a stable dose for at least 2 months. On the day of data acquisition, participants took their medications as prescribed.

#### TD Children

A total of 38 typically developing children were recruited to serve as the control group via an email sent to the university faculty and staff. Exclusion criteria included a history of psychiatric or developmental disorders (one child), current use of psychotropic medication, epilepsy, brain abnormalities, diagnosis of ASD in an immediate family member, a score above the ASD cutoff on the Social Responsiveness Scale (SRS), left-handedness (three children), no data for either of the two eye conditions (nine children), and noisy or insufficient EEG data (three children). This resulted in a pool of 22 children from which a final sample of 13 children (two females) was selected based on age, gender, and IQ matching with the ASD group. This TD group had a mean age of 10.4 years (*SD* = 1.4) and an age range between 8 years 0 months and 12 years 5 months.

### Procedure

Five minutes of resting-state EEG was collected in two eye conditions: EO and EC, with a short (2–5 min) break between each condition. The order in which participants performed these conditions was counterbalanced. For participants with ASD up to 10 min of each resting condition was recorded when possible to maximize the availability of artifact-free data. Participants were asked to remain still and awake and allow their minds to wander. To minimize eye movements during the EO condition, participants were asked to fixate on a white cross in the center of a black background on the computer screen. To minimize eye movements during the EC condition, participants were asked to close their eyes while fixated on the cross on the screen and keep their eyes in the same position as if still gazing at the fixation cross. Participants were seated an average of four feet from the monitor.

EEG data was monitored online during recording to ensure quality. If the experimenter noticed obvious deviations from study protocol (e.g., tapping fingers, clenching the jaw, raising eyebrows), the task was paused and the participant was given verbal feedback regarding adhering to instructions. During instances when participants failed to follow the instructions, data collection was stopped and the participants were reinstructed on the task before resuming data collection. Data collection was aborted if a participant required two or more task interruptions plus re-instructions and that participant was excluded from further data analysis (see exclusion criteria).

### EEG

Continuous EEG was recorded from the scalp at a sampling rate of 1000 Hz using a high-density array of 128 Ag/AgCl electrodes embedded in soft sponges (Geodesic Sensor Net, EGI, Inc., Eugene, OR, United States) using NetStation 5.3 software. Recording began following the adjustment of electrode impedances to below 50 kΩ. During online recording, data was referenced to vertex with onscreen filters set at 0.1–100 Hz.

#### Signal Processing

Continuous EEG data were preprocessed using the NetStation Waveform Tools software. (i) Data were down-sampled to 125 Hz to reduce computational load, (ii) bandpass filtered between 2 and 20 Hz, (iii) notch filtered to remove 60 Hz noise, (iv) segmented into 2 s epochs, (v) processed for artifacts using NetStation Waveform Tools algorithms, (vi) all epochs were manually inspected for artifacts and those containing obvious eye-blink, saccades, muscle noise, or non-physiological artifacts not already excluded by the artifact detection algorithms in NetStation were rejected. Bad channels were replaced using the spherical spline interpolation algorithm (Perrin et al., 1989). The data were then re-referenced to the average reference and baseline corrected. Data were exported from NetStation 5.3 for offline analysis in the MATLAB^®^ (Version 2018a, The Mathworks, Natick, MA, United States) toolbox EEGLab (Version 14.1.2.0b)^1^. EEG microstates were extracted using the EEGLAB plugin developed by Dr. Thomas Koenig.^2^

#### Microstate Analysis

Following the methods of Koenig et al., 2002, the first 10 artifact-free epochs for each condition were analyzed (Koenig et al., 2002). For each of these datasets, the global field power (GFP) curve, equivalent to the standard deviation of amplitude across the entire average-referenced electrode montage for each sample of time (Skrandies, 1990), was calculated. The topographic maps occurring at the peaks of the GFP curve were entered into a Topographic Atomize and Agglomerate Hierarchical Clustering (T-AAHC) algorithm to isolate four microstate clusters within each dataset. We *a priori* chose to extract four group-level microstate clusters in order to remain consistent with previous studies that report the four canonical microstates. The group-level microstate classes were then identified for both participant groups for the EO and EC conditions separately. These group-level maps were sorted according to the normative maps published in Koenig et al. (2002). The order of the individual participant microstate template maps was determined by the spatial correlation with the group-level microstate classes. The original EEG data of each participant for both resting-state conditions were re-expressed as the microstate class that they were most similar to with respect to the group level maps, as determined by Pearson’s correlation. From this time series, the microstate parameters such as global explained variance (GEV), duration, frequency, coverage, and GFP were computed from the maps in their center-specific configurations.

### Psychometric Measures

Autism Diagnostic Observation Schedule – 2 (ADOS-2) (Lord et al., 2012): The ADOS-2 is a semi-structured, play-based assessment to evaluate the core features of ASD. In addition to providing a score to measure against diagnostic thresholds, the score provided by the ADOS-2 is a marker of ASD severity (Gotham et al., 2009).

Social Communication Questionnaire (SCQ) (Rutter et al., 2003): The SCQ is a parent report questionnaire used to evaluate the communication skills and social functioning of children with ASD.

Social Responsiveness Scale (SRS) (Constantino and Gruber, 2002): The SRS is a parent report questionnaire intended to measure social behavior impairments such as social awareness, social information processing, capacity for reciprocal social communication, and social anxiety/avoidance in children between the ages of 4–18 years. An additional section of the SRS contains questions regarding preoccupations and traits related to autism.

Repetitive Behavior Scale – Revised (RBS-R) (Bodfish et al., 1999): The RBS-R is a parent report questionnaire that assesses five categories of repetitive behaviors, namely motor stereotypy, repetitive self-injury, compulsions, routines/sameness, and restricted interests.

Stanford Binet-5 Abbreviated IQ Test (SB5) (Roid, 2003): The Stanford Binet is an IQ test developed to measure developmental or intellectual disabilities in children. The five factors tested are knowledge, quantitative reasoning, visual-spatial processing, working memory, and fluid reasoning. The present study used the abbreviated version to obtain an estimate of each child’s developmental level.

### Statistical Analysis

Statistical analyses were performed in SPSS v. 26.0 (IBM Corp., Armonk, NY, United States). Separate repeated-measures (RM) analyses of variance (ANOVAs) were conducted for each microstate for the following parameters: GEV, duration, occurrence, coverage, and GFP. Each ANOVA contained one between-subject factor for group (ASD or TD), and one within-subject factor for eye condition (EC or EO). Significant interaction effects (group × eye condition) were decomposed using *post hoc* analyses to identify the direction of the effect. Statistical significance was set at a *p*-value less than 0.05 for the RM-ANOVAs. The significance levels of these *post hoc* comparisons were corrected for multiple comparisons using the Bonferroni method (statistically significant *p*-value set at 0.0125).

To assess the relationships between microstate measures and psychometric data in ASD, Pearson’s *r* was employed. To adjust for the skewness in the distribution of psychometric data, each variable was log transformed by a factor of log(*x*+1). To reduce the number of correlations examined, and in line with the *a priori* hypotheses of the study, the correlation analyses of microstate parameters with psychometric measures were restricted to examining microstate C frequency in the EC condition in ASD and age, IQ, SRS-Total, and RBSR-Total (with pairwise error rate corrected using the Bonferroni method).

## Results

**FIGURE 1:**
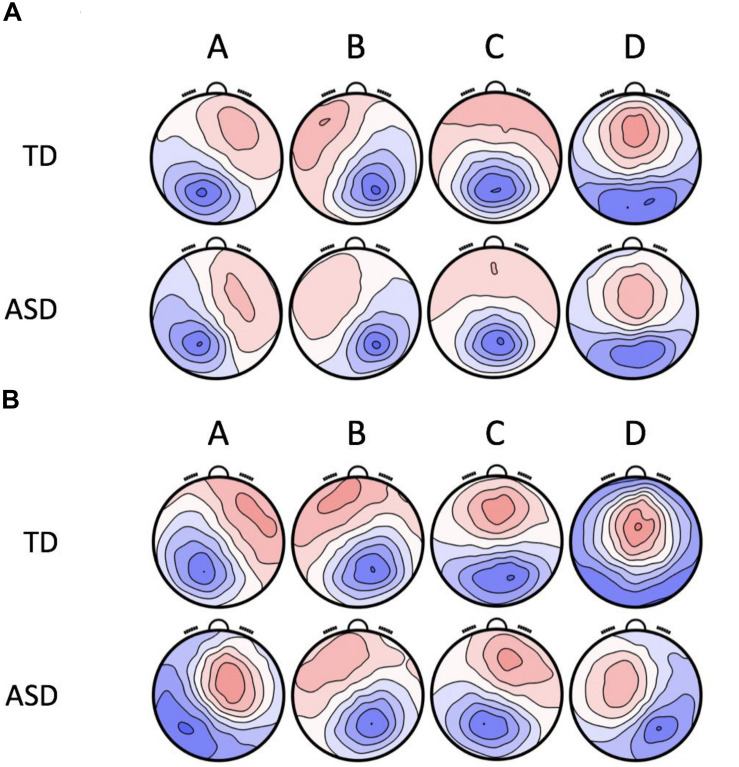
The group-level maps of the four microstate classes (A–D) in ASD and TD, independently computed for the two resting-state conditions. **(A)** Group Level Microstate Maps in Eyes Closed Resting-state. **(B)** Group Level Microstate Maps in Eyes Open Resting-state.

The topographies of the four microstate classes are displayed in Figure 1. The group level maps resemble those of the normative data (Koenig et al., 2002) and those reported in previous studies (Koenig et al., 2002; Britz et al., 2010). The four microstate classes were labeled A, B, C, and D, respectively, in accordance with their topographic shapes. Microstate A has a right frontal, left occipital orientation, Microstate B has a left frontal, right occipital orientation, Microstate C has an anterior-posterior orientation, and Microstate D has a fronto-central maximum. Table 2 shows the summary statistics for all the parameters of these microstates. The four microstate classes display different patterns in temporal dynamics across the two groups and eye conditions. These differences were statistically tested using RM-ANOVAs. The results of the RM-ANOVAs are displayed in Table 3.

**TABLE 2:** Descriptive statistics of microstate parameters from EC and EO conditions.

	**Microstate**	**Eyes-Closed**		**Eyes-Open**	
		**TD**	**ASD**	**TD**	**ASD**
		**Mean (SD)**	**Mean (SD)**	**Mean (SD)**	**Mean (SD)**
GEV		73.23 (4.42)	77.68 (3.59)	72.88 (5.00)	75.08 (3.26)
Duration (ms)	A	73.58 (11.53)	81.32 (12.75)	73.20 (9.07)	75.75 (13.17)
	B	69.32 (9.91)	83.87 (13.08)	70.01 (11.23)	73.63 (11.56)
	C	67.32 (9.27)	81.56 (16.67)	66.99 (9.92)	69.28 (10.72)
	D	63.99 (11.10)	80.67 (15.32)	63.35 (8.24)	68.56 (8.03)
Frequency (Hz)	A	3.87 (0.41)	3.39 (0.67)	4.13 (0.60)	3.76 (0.46)
	B	3.87 (0.62)	3.43 (0.64)	4.06 (0.46)	3.86 (0.61)
	C	4.04 (0.55)	3.24 (0.42)	3.85 (0.51)	3.70 (0.43)
	D	4.04 (0.58)	3.46 (0.51)	3.88 (0.59)	4.04 (0.47)
Coverage (%)	A	26.21 (3.97)	24.84 (4.85)	27.76 (3.88)	25.52 (2.82)
	B	25.02 (4.43)	26.07 (4.02)	26.22 (4.10)	25.69 (2.62)
	C	24.94 (4.81)	24.29 (4.06)	23.29 (3.00)	23.71 (3.27)
	D	23.82 (4.12)	24.79 (4.31)	22.73 (3.03)	25.08 (2.74)
GFP	A	11.57 (2.71)	6.83 (2.19)	8.87 (2.09)	5.19 (1.09)
	B	11.38 (2.54)	7.35 (2.13)	8.83 (2.12)	5.46 (1.03)
	C	11.60 (2.73)	7.52 (2.21)	8.90 (2.10)	5.65 (1.06)
	D	11.60 (2.75)	7.37 (2.16)	8.74 (2.10)	5.53 (1.14)

**TABLE 3:** Results of RM-ANOVAs.

	***df***	***F***	**Sig.**	**Partial eta squared**	**Observed power**		**df**	**F**	**Sig.**	**Partial eta squared**	**Observed power**

**Duration**	**Microstate A**					**Duration**	**Microstate B**				
Eye Condition × Group	1, 24	0.910	0.350	0.037	0.150	Eye Condition × Group	1, 24	5.417	**0.029**	0.184	0.608
Eye Condition	1, 24	1.195	0.285	0.047	0.183	Eye Condition	1, 24	4.134	0.053	0.147	0.497
Group	1, 24	1.942	0.176	0.075	0.267	Group	1, 24	5.571	**0.027**	0.188	0.620

**Frequency**						**Frequency**					

Eye Condition × Group	1, 24	0.187	0.669	0.008	0.070	Eye Condition × Group	1, 24	0.905	0.351	0.036	0.150
Eye Condition	1, 24	5.605	**0.026**	0.189	0.623	Eye Condition	1, 24	6.258	**0.020**	0.207	0.670
Group	1, 24	6.391	**0.018**	0.210	0.679	Group	1, 24	2.817	0.106	0.105	0.364

**Coverage**						**Coverage**					

Eye Condition × Group	1, 24	0.164	0.689	0.007	0.067	Eye Condition × Group	1, 24	0.714	0.406	0.029	0.128
Eye Condition	1, 24	1.058	0.314	0.042	0.167	Eye Condition	1, 24	0.188	0.669	0.008	0.070
Group	1, 24	2.653	0.116	0.100	0.346	Group	1, 24	0.048	0.829	0.002	0.055

**GFP**						**GFP**					

Eye Condition × Group	1, 21	3.143	0.091	0.130	0.394	Eye Condition × Group	1, 22	1.405	0.249	0.060	0.205
Eye Condition	1, 21	52.294	**0.000**	0.713	1.000	Eye Condition	1, 22	61.996	**0.000**	0.738	1.000
Group	1, 21	24.373	**0.000**	0.537	0.997	Group	1, 22	22.516	**0.000**	0.506	0.995

**Duration**	**Microstate C**					**Duration**	**Microstate D**				

Eye Condition × Group	1, 24	7.305	**0.012**	0.233	0.737	Eye Condition × Group	1, 24	4.538	**0.044**	0.159	0.534
Eye Condition	1, 24	8.129	**0.009**	0.253	0.781	Eye Condition	1, 24	5.609	**0.026**	0.189	0.623
Group	1, 24	4.118	0.054	0.146	0.495	Group	1, 24	10.311	**0.004**	0.301	0.869

**Frequency**						**Frequency**					

Eye Condition × Group	1, 24	12.248	**0.002**	0.338	0.919	Eye Condition × Group	1, 24	11.291	**0.003**	0.320	0.897
Eye Condition	1, 24	2.069	0.163	0.079	0.282	Eye Condition	1, 24	3.683	0.067	0.133	0.453
Group	1, 24	8.279	**0.008**	0.256	0.788	Group	1, 24	1.254	0.274	0.050	0.189

**Coverage**						**Coverage**					

Eye Condition × Group	1, 24	0.324	0.574	0.013	0.085	Eye Condition × Group	1, 24	0.523	0.476	0.021	0.107
Eye Condition	1, 24	1.387	0.251	0.055	0.204	Eye Condition	1, 24	0.180	0.676	0.007	0.069
Group	1, 24	0.009	0.923	0.000	0.051	Group	1, 24	2.481	0.128	0.094	0.327

**GFP**						**GFP**					

Eye Condition × Group	1, 24	2.025	0.168	0.078	0.277	Eye Condition × Group	1, 23	3.331	0.081	0.126	0.416
Eye Condition	1, 24	63.149	**0.000**	0.725	1.000	Eye Condition	1, 23	69.557	**0.000**	0.752	1.000
Group	1, 24	22.234	**0.000**	0.481	0.995	Group	1, 23	21.391	**0.000**	0.482	0.993

*Significant *p*-values are bolded.*

### GEV

The total GEV is a measure of how well all four microstate template maps together explain the topographies for every moment in time for each dataset (Murray et al., 2008). There were no significant interactions between the group × eye condition for GEV [*F*(1,24) = 3.175, *p* = 0.088, $\eta_{p}^{2}$ = 0.117]. There was a main effect of group [*F*(1,24) = 4.981, *p* = 0.035, $\eta_{p}^{2}$ = 0.172] with greater GEV in ASD (*p* = 0.005) and a main effect of eye condition [*F*(1,24) = 5.450, *p* = 0.028, $\eta_{p}^{2}$ = 0.185]. However, the GEV for the two conditions was not significantly different after correcting for multiple comparisons (*p* = 0.233).

### Microstate A

There were no significant interactions between group × eye condition for duration, frequency, coverage, or GFP. There was a main effect of eye condition for frequency of microstate A [*F*(1,24) = 5.605, *p* = 0.026, $\eta_{p}^{2}$ = 0.189] with increased frequency of microstate A in EO (*p* = 0.012). There were no main effects of group or eye condition for duration and coverage. For the GFP of microstate A, there was a main effect of group [*F*(1,24) = 24.3, *p* < 0.0001, $\eta_{p}^{2}$ = 0.537] with decreased GFP in ASD (*p* < 0.0001) and a main effect of eye condition [*F*(1,24) = 52.3, *p* < 0.0001, $\eta_{p}^{2}$ = 0.713] with decreased GFP in the EO condition (*p* = 0.012).

### Microstate B

**FIGURE 2:**
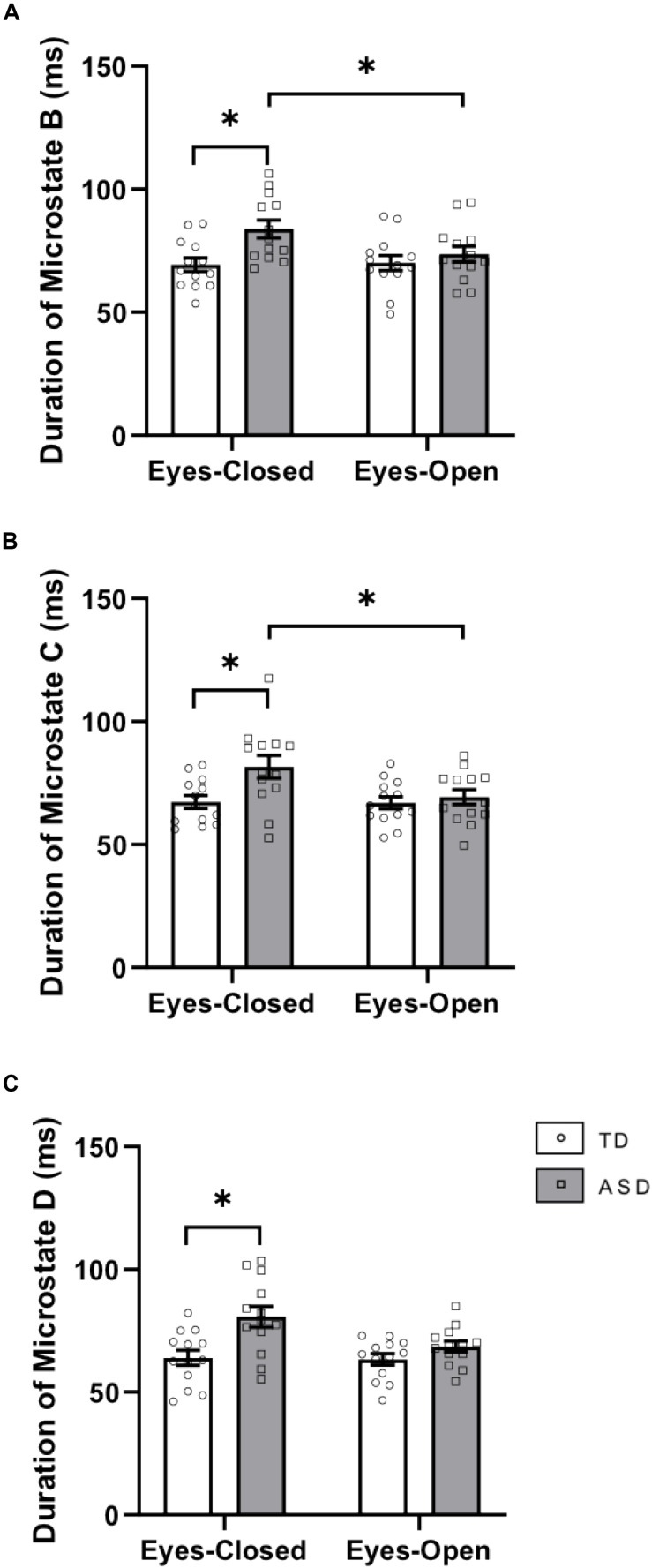
Durations of **(A)** microstate class B, **(B)** microstate class C, and **(C)** microstate class D in EC and EO for the two groups. This graph displays the mean duration of each microstate with corresponding standard error of the mean. The white and gray bars display results from the TD and ASD groups, respectively. individual participant data is plotted on each bar. All significant differences are indicated with asterisks (*p* < 0.0125, Bonferroni corrected).

There was a significant interaction between group × eye condition for duration [*F*(1,24) = 5.417, *p* = 0.029, $\eta_{p}^{2}$ = 0.184]. *Post hoc* tests showed that within the ASD group, the duration of microstate B was greater in EC (*p* = 0.0051) than in EO (Figure 2A). Across the groups, the duration of microstate B was greater in ASD than in TD in the EC condition (*p* = 0.0039). There was a main effect of group for duration [*F*(1,24) = 5.571, *p* = 0.027, $\eta_{p}^{2}$ = 0.188] with increased duration of microstate B in ASD (*p* = 0.008). There was no main effect of eye condition for the duration of microstate B.

There were no significant interactions between group × eye condition for frequency, coverage, or GFP. There was a main effect of eye condition for frequency [*F*(1,24) = 6.258, *p* = 0.020, $\eta_{p}^{2}$ = 0.207] with a trend toward increased frequencies of microstate B in EO (*p* = 0.064). For the GFP of microstate B, there was a main effect of group [*F*(1,24) = 22.5, *p* < 0.0001, $\eta_{p}^{2}$ = 0.506] with decreased GFPs in ASD (*p* < 0.0001) and a main effect of eye condition [*F*(1,24) = 61.9, *p* < 0.0001, $\eta_{p}^{2}$ = −0.738] with decreased GFP in the EO condition (*p* = 0.006).

### Microstate C

There was a significant interaction between group × eye condition for duration [F(1,24) = 7.305, *p* = 0.012, $\eta_{p}^{2}$ = 0.233]. *Post hoc* tests showed that within the ASD group, the duration of microstate C was greater in EC (*p* = 0.0006) than in EO (Figure 2B). Across the groups, the duration of microstate C was greater in ASD than in TD in the EC condition (*p* = 0.01275). There was a main effect of eye condition for the duration of microstate C [*F*(1,24) = 8.129, *p* = 0.009, $\eta_{p}^{2}$ = 0.253] with a trend toward greater duration of microstate C in EC (*p* = 0.079).

**FIGURE 3:**
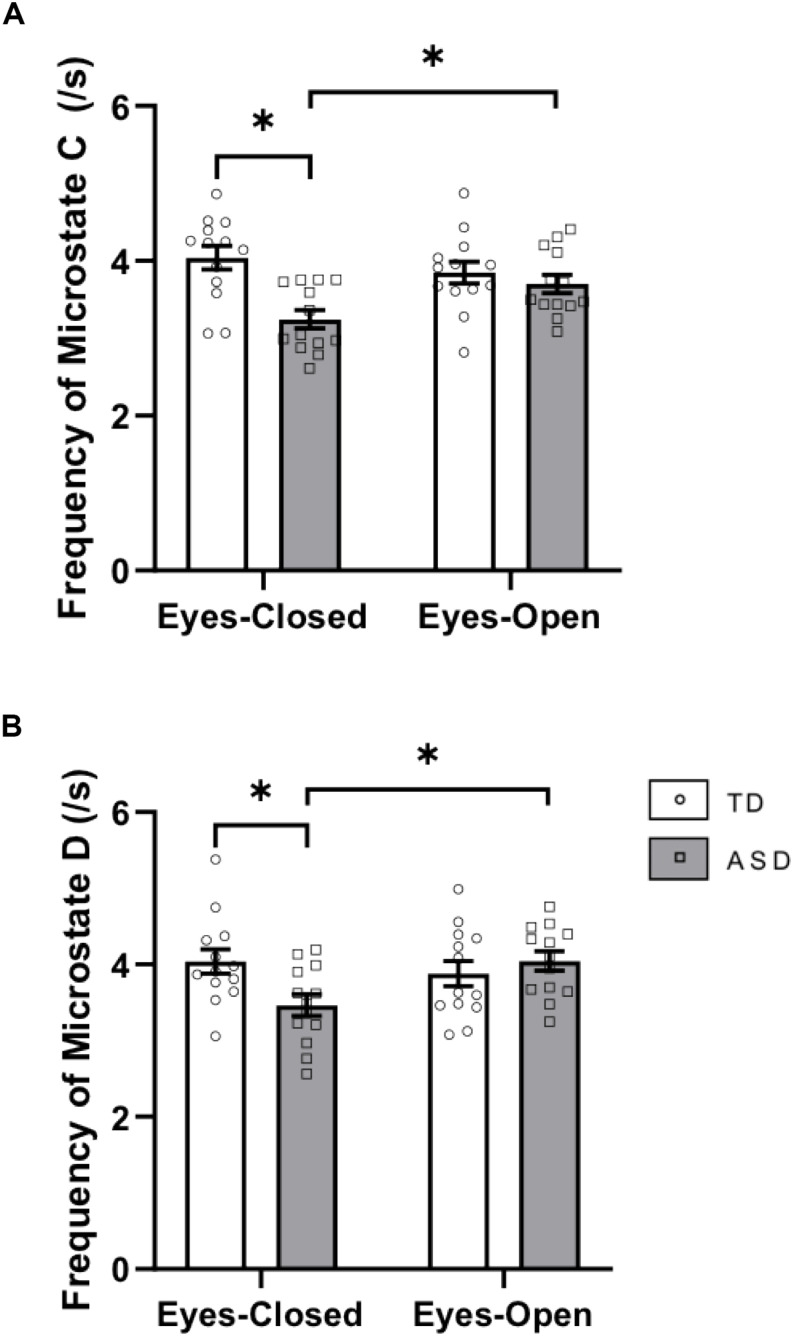
Frequency of **(A)** microstate class C and **(B)** microstate class D in EC and EO for the two groups. These graphs display the mean frequencies of each microstate with corresponding standard error of the mean. The white and gray bars display results from the TD and ASD groups, respectively. individual participant data is plotted on each bar. All significant differences are indicated with asterisks (*p* < 0.0125, Bonferroni corrected).

There was a significant interaction between group × eye condition for frequency [*F*(1,24) = 12.248, *p* = 0.002, $\eta_{p}^{2}$ = 0.338]. *Post hoc* tests showed that within the ASD group, microstate C was less frequent in EC (*p* = 0.0019) than in EO. Across the groups, microstate C was less frequent in ASD than in TD in the EC condition (*p* = 0.0003) (Figure 3A). There was a main effect of group for the frequency of microstate C [*F*(1,24) = 8.279, *p* = 0.008, $\eta_{p}^{2}$ = 0.256] with decreased frequencies of microstate C in ASD (*p* = 0.0014).

There were no significant interactions between group × eye condition for coverage. For the GFP of microstate C, there was a main effect of group [*F*(1,24) = 22.2, *p* < 0.0001, $\eta_{p}^{2}$ = 0.481] with decreased GFP in ASD (*p* < 0.0001) and a main effect of eye condition [*F*(1,24) = 63.1, *p* < 0.0001, $\eta_{p}^{2}$ = 0.725] with decreased GFP in the EO condition (*p* = 0.0049).

### Microstate D

There was a significant interaction between group × eye condition for duration [*F*(1,24) = 4.538, *p* = 0.044, $\eta_{p}^{2}$ = 0.159]. *Post hoc* tests showed that within the ASD group, the duration of microstate D was greater in EC (*p* = 0.0040) than in EO. Across the groups, microstate D had a greater duration in ASD than in TD in the EC condition (*p* = 0.0040). There was a main effect of eye condition for the duration of microstate D [*F*(1,24) = 5.609, *p* = 0.026, $\eta_{p}^{2}$ = 0.189] with a trend toward greater duration of microstate D in EC (*p* = 0.073). There was also a main effect of group for the duration of microstate D [*F*(1,24) = 10.3, *p* = 0.004, $\eta_{p}^{2}$ = 0.301] with a greater duration of microstate D in ASD (*p* = 0.001) (Figure 2C).

There was a significant interaction between group × eye condition for frequency [*F*(1,24) = 11.291, *p* = 0.003, $\eta_{p}^{2}$ = 0.320]. *Post hoc* tests showed that within the ASD group, microstate D was less frequent in EC than in EO (*p* = 0.0010). Across the groups, microstate D trended toward being less frequent in ASD than in TD in EC (*p* = 0.0135) (Figure 3B). There were no main effects of group or eye condition for frequency of microstate D.

There were no significant interactions between group × eye condition for coverage [*F*(1,24) = 0.523, *p* = 0.476, $\eta_{p}^{2}$ = 0.021] or GFP [*F*(1,24) = 3.331, *p* = 0.081, $\eta_{p}^{2}$ = 0.126]. For the GFP of microstate D, there was a main effect of group [*F*(1,24) = 22.2, *p* < 0.0001, $\eta_{p}^{2}$ = 0.482] with decreased GFP in ASD (*p* < 0.0001) and a main effect of eye condition [*F*(1,24) = 63.1, *p* < 0.0001, $\eta_{p}^{2}$ = 0.752] with decreased GFP in the EO condition (*p* = 0.0039).

### Correlations of Microstate Parameters and Psychometric Measures

No significant correlation was found between microstate C frequency and these psychometric measures with and without correction using the Bonferroni method for multiple comparisons.

## Discussion

The purpose of this study was to investigate the resting-state EEG microstate architecture in ASD under EC and EO conditions. Based on established findings for SN activity in ASD using fMRI, we hypothesized that the temporal dynamics of microstate C would be altered in ASD in both eye conditions reflecting atypical activity. ASD and TD children between the ages of 8–14 years performed two resting-state tasks for 5 min, keeping their eyes either open or closed during data collection. The main outcomes of this study were that children with ASD produced topographically similar canonical microstates at rest in comparison to their TD peers. The differences between these groups in the parameters of these microstates were largely restricted to the eyes closed resting-state condition.

The four microstate maps (labeled A, B, C, and D) isolated for both resting-state conditions in the two groups were topographically similar to the canonical maps described in the normative study of adults by Koenig et al., 2002. In the EC resting-state, they accounted for 73.2 and 72.8% of the explained variance in TD and ASD, respectively. In the EO resting-state, these microstate maps accounted for 77.7 and 75.0% of the explained variance in TD and ASD, respectively. According to prior studies (Britz et al., 2010), microstate A reflects the activity of the bilateral superior temporal and parietal cortex, interpreted as the auditory cortex, microstate B reflects the activity of the striate and extrastriate cortex or the visual network, microstate C reflects the activity of the bilateral insular and anterior cingulate cortex or the SN, and microstate D reflects the activity of the fronto-parietal attentional network.

The findings of this study supported our hypothesis that the parameters of microstate C are altered in ASD at rest. This hypothesis was based on fMRI findings which described aberrant SN activity in ASD (Supekar et al., 2013; Nomi and Uddin, 2015; Abbott et al., 2016; Green et al., 2016; Chen H. et al., 2017; Damiani et al., 2018) and numerous studies that have linked microstate C to the SN (Stevens et al., 1997). Functionally, the SN has been presumed to have two roles: (1) a bottom-up process for the extraction of salient sensory stimuli from the external or internal environment and (2) a top-down mechanism for focusing one’s spotlight of attention on a goal-directed behavior (Menon and Uddin, 2010). In this study, at rest in the EC condition, we found that the duration of microstate C was increased, and the frequency of microstate C was decreased in children with ASD as compared to TD. Functionally, in ASD, this would imply that the SN remains activated for longer durations and is activated less frequently. This may explain the difficulty in processing complex but subtle environmental stimuli such as social cues in ASD individuals. Clinically, these difficulties may lead to impaired social and communication skills. We also found that the changes in the microstate parameters in ASD were not restricted to microstate C. The durations of microstates B and D were also increased in ASD in EC, which is consistent with the finding of longer dwell times across all brain networks in children with ASD using fMRI (Dierks et al., 1997; Atluri et al., 2017). This is also consistent with various reports of hyper-connectivity across brain networks using fMRI in ASD at the whole brain level (Chen S. et al., 2017; Damiani et al., 2018). Similarly, the frequencies of microstates A and B were significantly decreased in ASD in EC. This is consistent with findings in fMRI studies showing decreased switching between global brain networks in autism (Chen H. et al., 2017).

Our findings showing altered microstate dynamics in ASD are similar to observations by Jia and Yu (2019). However, the specific group differences were not the same. Whereas the duration of microstates C and A were significantly reduced in ASD compared to TD in the previous study, we found increased duration of microstate C as well as microstates B and D. Similarly, the previous study reported an increase in the frequency of microstate B in ASD as compared to TD, whereas we found that the frequencies of microstates A, B, and C were significantly reduced in ASD as compared to TD. The differences in microstate dynamics between these two studies may be explained by differences in the data collection methods as well as differences in the age range of the participants included. Whereas the previous study combined EC and EO resting-state data, we collected data from these two conditions as separate instances and analyzed them independently. This was based on a previous study by Seitzman et al., 2017 that found differences in EC and EO resting-state microstate dynamics, and also on several previous fMRI resting-state studies that have reported differences in network activity across resting-state conditions. Consistent with Seitzman et al., 2017, we found that the frequency of microstate B was increased and the durations of microstates A and D were decreased in the EO condition across groups.

In this study, we found that ASD-related differences in EEG microstate architecture were largely restricted to the EC resting-state condition. fMRI studies have suggested differences in the topological organizations of functional neural networks for exteroceptive and interoceptive processing during EO and EC resting-states (Xu et al., 2014). These studies observed an increase in interoceptive and self-referential processes in the EC condition as compared to the EO condition, which is reflected by an increase in the activity of the default mode network (DMN) (Marx et al., 2004; Xu et al., 2014). In addition to the SN, microstate C may also represent the activity of the anterior DMN (Britz et al., 2010; Milz et al., 2016; Michel and Koenig, 2018), which is implicated in self-referential processes as well as the integration of interoceptive information with emotional salience to form a subjective representation of a subject’s own body (Taylor et al., 2009; Andrews-Hanna, 2012). The group differences in microstate C parameters observed in the EC resting-state may be a result of differences in these interoceptive processes in ASD. Several studies have suggested an increased attention to internal rather than external cues in ASD (Schauder et al., 2015; Noel et al., 2018). During the EO resting-state, the differences in microstate parameters between the two groups may no longer be apparent due to the suppression of these interoceptive processes as resources are allocated to exteroceptive processes. The neural generators of microstate C are an area of active debate and require further exploration (Michel and Koenig, 2018).

These findings should be interpreted within the framework of the limitations of this study. One limitation is the amount of data used for the microstate analysis. We chose 20 s (10 epochs) of artifact-free data across participants for each of the resting-state conditions. This same amount of data was used by Koenig et al., 2002 for the generation of the normative microstate data and has been shown to be sufficient for this type of analysis (Lehmann et al., 2005; Nishida et al., 2013; Michel and Koenig, 2018). Other studies report larger amounts of data for microstate analysis per participant. However, those studies often employ artifact correction pipelines that may alter the underlying structure of the data. In contrast, our data were not modified in any manner. Another limitation of this study is that not all ASD participants were able to complete both resting-state conditions. Hence, the final sample size was considerably reduced. However, it also allowed us to systematically compare two functionally different resting-states in EC and EO conditions, which was not controlled for in the prior ASD study (Jia and Yu, 2019). Data loss in the ASD group necessitated reduction in the TD sample to ensure that the two participant groups remained closely matched at an individual level and to reduce the possibility that observed group differences in microstates could be explained by age, gender or IQ differences. Re-analysis of the full TD sample recapitulated the differences in microstate parameters observed for the smaller subgroup (Supplementary Figure S1 and Supplementary Tables S1, S2). Another potential limitation of our study is that we *a priori* chose to focus on four canonical microstate clusters for our analyses instead of applying a more recently described meta-criterion to generate a data-specific number of optimal clusters. The latter method resolves microstate C into two separate clusters that are thought to independently represent the SN and the DMN. However, it is still unclear if using the meta-criterion across different datasets of the same populations generates similar cluster numbers. Furthermore, using the four canonical clusters allowed us to directly compare our findings to other studies in ASD populations. We expected a correlation between the dynamics of the SN (as represented by microstate C) and the severity of ASD behaviors. However, the absence of the predicted association may reflect a need for a more nuanced functional interpretation of microstate C. Recent findings (Custo et al., 2017; Seitzman et al., 2017) suggest that it may also represent the activity of the DMN. Future studies resolving microstate C into two separate clusters for SN and DMN may help uncover their associations with the psychometric measures and address this limitation. Finally, we did not use topographic ANOVA to determine the spatial differences between the group level microstates. The group level microstate maps in the EO condition of ASD neither resemble the EC condition of ASD nor the group level maps of TD. Given that the topographies are not visually similar, the underlying networks generating these patterns can be assumed to be different from the EC condition (Murray et al., 2008). It is necessary to further investigate this finding using methods such as source localization. This could potentially explain why we were not able to detect meaningful differences between the two groups in the EO condition.

In conclusion, the current study provided preliminary evidence for alterations in EEG microstate architecture in ASD as compared to TD children, providing an understanding of the potential underlying etiology of ASD behavior in terms of brain network dynamics. These findings are consistent with previous studies in ASD using fMRI and thus add to the literature on brain network dynamics.

## Data Availability Statement

The raw data supporting the conclusions of this article will be made available by the authors, without undue reservation.

## Ethics Statement

The studies involving human participants were reviewed and approved by Vanderbilt University Institutional Review Board. Written informed consent to participate in this study was provided by the participants’ legal guardian/next of kin.

## Author Contributions

SNK and JB contributed to the conception of the study and critically revised the manuscript and provided crucial feedback. SNK and AW recruited participants and collected data. SNK performed the pre-processing of the data, microstate analysis, statistical tests, and wrote the draft of the manuscript. AK provided expertise on EEG analyses, pre-processing, and the functional interpretation of the results. JB provided expertise on autism spectrum disorders and the interpretation of results. All authors contributed to the article and approved the submitted version.

## Conflict of Interest

The authors declare that the research was conducted in the absence of any commercial or financial relationships that could be construed as a potential conflict of interest.

## Footnotes

1 http://sccn.ucsd.edu/eeglab/

2 http://www.thomaskoenig.ch/index.php/software
