# Supplementary material for: Children With Autism Produce a Unique Pattern of EEG Microstates During an Eyes Closed Resting-State Condition
Source: Front Hum Neurosci. 2020 Oct 8;14:288. doi: 10.3389/fnhum.2020.00288 (PMC7579608; doi:10.3389/fnhum.2020.00288)
Supplement: Supplementary file 1 [file Data_Sheet_1.pdf]

Supplementary data

Re-analysis of the full TD sample recapitulated the differences in microstate parameters observed for the smaller subgroup. For example, the differences in microstate frequency observed between EC and EO in the larger TD group of 22 participants also replicated the findings of Seitzman *et al.*, 2017.

A RM-ANOVA was performed with the two factors of eye-condition and microstates to compare the differences in microstate frequency in the larger TD group between the EC and EO conditions. The condition x microstate interaction was not significant for frequency (F (3, 63) = 0.66, p = 0.583, $\eta_p^2 = 0.03$). The main effect of condition was significant (F (1, 21) = 19.39, p = 0.0002, $\eta_p^2 = 0.480$), with microstates occurring more frequently in the eyes-open condition as compared to the eyes-closed condition. On average, microstate A (p = 0.0094) and B (p = 0.0038) occurred more frequently in the eyes-open condition than in the eyes-closed condition after Bonferroni correction. There was a significant main effect of microstate class (F (3, 63) = 4.16, p = 0.009, $\eta_p^2 = 0.165$) with microstate B occurring more frequently than microstate A in the eyes-open condition (p = 0.032), after Bonferroni correction. The results are displayed in Figure S1. The means and standard deviations of microstate frequency during both resting states are shown in Table S1 and the results of the RM-ANOVA are displayed in Table S2.

Figure S1: Frequency of microstates for all 22 TD participants.

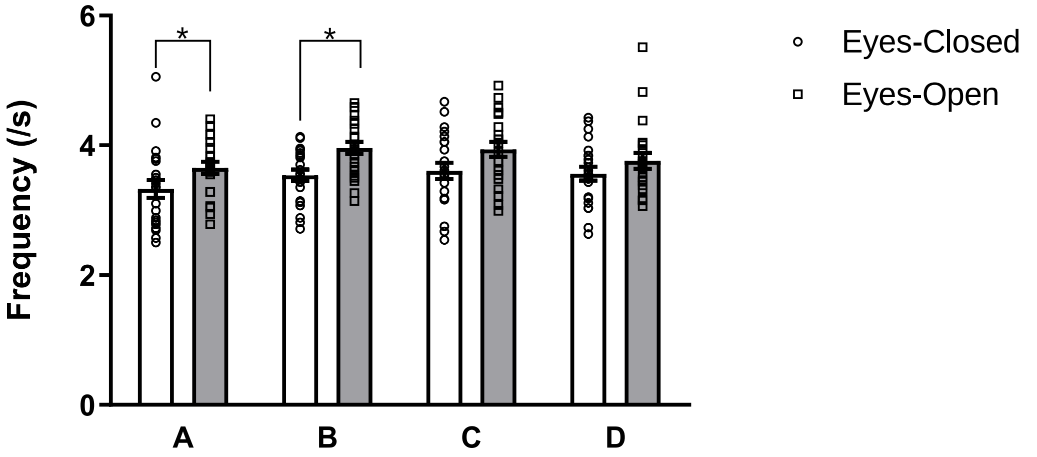

Table S1: Descriptive statistics of microstate frequency for all 22 TD participants from EC and EO conditions.

	**Eyes-Closed Mean (SD)**	**Eyes-Open Mean (SD)**
**A**	3.33 (0.63)	3.65 (0.44)
**B**	3.54 (0.43)	3.96 (0.44)
**C**	3.61 (0.59)	3.94 (0.55)
**D**	3.57 (0.5)	3.76 (0.58)

Table S2: Results of RM-ANOVA for frequency of eye-condition by microstate for frequency of all 22 TD participants. Significant p-values are bolded.

	**df**	**F**	**p-value**	**Partial Eta Squared**
**Eye-Condition**	1, 21	19.39	**0.0002**	0.48
**Microstate**	3, 63	4.16	**0.009**	0.165
**Interaction**	3, 63	0.66	0.583	0.03
